# A portable near infrared spectroscopy system for bedside monitoring of newborn brain

**DOI:** 10.1186/1475-925X-4-29

**Published:** 2005-04-29

**Authors:** Alper Bozkurt, Arye Rosen, Harel Rosen, Banu Onaral

**Affiliations:** 1 School of Biomedical Engineering, Science and Health Systems, Drexel University, 3141 Chestnut Street Philadelphia, Pennsylvania 19104, USA; 2 St.Peter's University Hospital, 254 Easton Ave, New Brunswick, New Jersey 08801, USA

## Abstract

**Background:**

Newborns with critical health conditions are monitored in neonatal intensive care units (NICU). In NICU, one of the most important problems that they face is the risk of brain injury. There is a need for continuous monitoring of newborn's brain function to prevent any potential brain injury. This type of monitoring should not interfere with intensive care of the newborn. Therefore, it should be non-invasive and portable.

**Methods:**

In this paper, a low-cost, battery operated, dual wavelength, continuous wave near infrared spectroscopy system for continuous bedside hemodynamic monitoring of neonatal brain is presented. The system has been designed to optimize SNR by optimizing the wavelength-multiplexing parameters with special emphasis on safety issues concerning burn injuries. SNR improvement by utilizing the entire dynamic range has been satisfied with modifications in analog circuitry.

**Results and Conclusion:**

As a result, a shot-limited SNR of 67 dB has been achieved for 10 Hz temporal resolution. The system can operate more than 30 hours without recharging when an off-the-shelf 1850 mAh-7.2 V battery is used. Laboratory tests with optical phantoms and preliminary data recorded in NICU demonstrate the potential of the system as a reliable clinical tool to be employed in the bedside regional monitoring of newborn brain metabolism under intensive care.

## I. Background

When light in the near infrared (NIR) range of the spectrum is shone through the scalp, injected photons follow various paths inside the head. Some of these photons are absorbed by different layers of the tissue such as skin, skull and brain. Others exit the head after following the so-called "banana" pattern due to scattering effect of the tissue [1]. Backscattered photons can be detected by means of appropriate optical apparatus. When the absorption spectrum of light is analyzed, it is seen that the main absorbers in the NIR range are blood chromophores of oxygenated and deoxygenated hemoglobin (HbO_2 _and Hb, respectively). Water and lipid are relatively transparent to NIR light. Therefore, changes in the amplitude of backscattered light can be interpreted as changes in blood chromophore concentrations. The procedure of estimating blood chromophore concentrations by means of near infrared light is called Near Infrared Spectroscopy (NIRS)[1]. Blood chromophore information can be used to estimate blood volume and tissue oxygenation which are indications of hemodynamic activity.

Different approaches can be used to implement NIRS such as time resolved, frequency domain and continuous wave techniques [2]. Among these methods, continuous wave (cw) NIRS is the most practical one, where light with constant amplitude is injected to tissue and amplitude decay of the light intensity due to absorption is analyzed. Changes in light amplitude are used to calculate changes in concentrations of blood chromophores. Due to its practicality cwNIRS systems allow for bedside or home monitoring of blood chromophores for extended periods.

Pulse oximetry, a NIR light based technique similar to NIRS, is being widely used in current clinical practice. The aim of pulse oximetry is to detect arterial blood saturation. CwNIRS further expands the application window of NIR light by providing information about blood dynamics in capillaries. Although, the arterial saturation obtained via pulse oximeter can only provide global information about the clinical state of the patient, capillary blood dynamics studied with cwNIRS is capable of supplying local tissue oxygenation information which can be used for various clinical purposes.

One of the important and promising application areas of cwNIRS technique is clinical neonatology. CwNIRS systems are non-invasive and low-power systems. They allow real time measurements without removing newborns from nursery units, thereby not interfering with intensive care. This is especially important for the vulnerable preterm neonate population. Some clinical situations, where neonatal cwNIRS can be employed, are asphyxia, hypoglycemia, apnea, endotracheal suctioning, aminophylline administration, indomethacin and exogenous surfactant administrations [3]. By spanning such a large application space, cwNIRS has a strong potential to be an inevitable part of clinical neonatalogy by monitoring cerebral hemodynamics of pre- and full-term newborns. Until now, a number of NIRS systems have been reported by various groups to assess oxygenation related changes on infant brain [4-7]. Various clinical studies have been run by using these systems [8-13].

In this paper, we present and discuss design issues of a portable, battery-operated, low-cost, low-noise, fast cwNIRS system which has been designed for bedside cerebral hemodynamic monitoring of newborns in various clinical studies. To our knowledge, none of the reported NIRS systems provide these features taken together, which is a crucial factor in transferring the technology to NICU for continuous bedside clinical monitoring, particularly, of regional brain metabolism. Moreover, the system provides access to raw photon data coming from detectors for further off-line signal processing. There are a number of open biomedical signal processing questions such as removing physiological noise and motion artifacts from NIRS data as well as hemodynamic detection of evoked events which are still waiting to be explored by biomedical signal processing experts.

As a preliminary evaluation, we present pilot results demonstrating that the system is capable of monitoring changes in HbO_2 _and Hb concentrations in Intralipid solution and in infant brain.

## II. Methods

### A. Principles of cwNIRS Measurement Device

The change in the amount of blood chromophores in the tissue can be predicted by means of Modified Beer Lambert Law as explained below [14,15].

**Figure 1:**
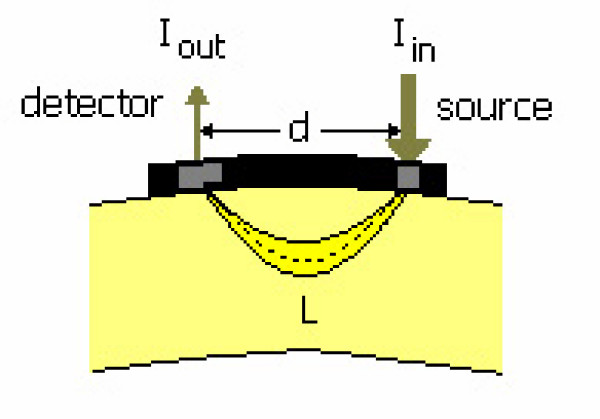
Propagation of photons between source and detector and parameters for Modified Beer Lambert Law



When the NIR light source and detector are located as in fig 1, the detector receives backscattered photons. Some of the injected photons are lost as a result of scattering and absorption due to different structures in the tissue. The attenuation of light between the source and detector can be formulated as follows:

where I_in _is incident light, I_out _is the detected light and OD_*λ *_is the optical density for wavelength *λ*. Therefore, OD_*λ *_can be defined as attenuation in intensity of light as a function of wavelength *λ*. This attenuation is the superposition of absorption (A_*λ*_) and scattering (S_*λ*_) of light with wavelength *λ*. Thus,



Blood chromophores HbO_2 _and Hb are the main absorbers of light in the NIR region of the spectra. Therefore, absorption of light can be formulated as:



where *ε*_*i*,*λ *_is the specific extinction coefficient of blood chromophore for wavelength *λ*, C_i _is the concentration of blood chromophores and L_*λ *_is the pathlength of light at *λ*. Pathlength can be expressed in terms of source detector separation as:

*L*_*λ *_= *d*·*DPF*_*λ *_    (4)

Where d is the distance between the light source and the detector and DPF_*λ *_is the differential pathlength factor. The differential pathlength factor is the correction in the mean photon pathlength for scattering and defined as [16]:



where *μ*_*a*,*λ *_is the absorption coefficient and *μ*_*s'*,*λ *_is the reduced scattering coefficient at wavelength *λ*. Here, the reduced scattering coefficient is used instead of scattering coefficient due to the anisotropy of the scattering.

When scattering, therefore the differential pathlength factor, is assumed to be constant, two successive measurements yield the differential OD value of:



where the effect of scattering is cancelled.

Since each chromophore has a specific extinction coefficient and differential pathlength factor, measurement with two wavelengths leads to:



where

 and



 and  are defined as  and .

Therefore,



Equation 9 provides a transformation from light output change to change in blood chromophore concentrations. By using blood chromophore concentrations we define two parameters, namely,

*OXY *= Δ*C*_*HbO*2 _- Δ*C*_*Hb *_    (10)

and

*BV *= Δ*C*_*HbO*2 _+ Δ*C*_*Hb *_    (11)

OXY and BV are estimates proportional to, respectively, oxygenation and blood volume changes in the tissue due to hemodynamic activation.

#### Calculation of parameters

Absorption and reduced scattering coefficients cannot be measured with cwNIRS method, therefore, are calculated by using the values given in the literature[17,18]. An assumption of 85% saturation and 100 *μ*M total hemoglobin concentration[19] results in the absorption and reduced scattering coefficients of .

By using these coefficients in equation 5, DPF values are calculated as 5.0055 and 4.6564 for 730 nm and 850 nm, respectively. These values are consistent with the measurements in the literature[20]. On the other hand,  is obtained from the values given in the literature[15] as:



By inserting  into equation 8, the relationship between light intensity change and blood chromophore concentration is derived as:



### B. cwNIRS Instrumentation

#### a. Description of system design

**Figure 2:**
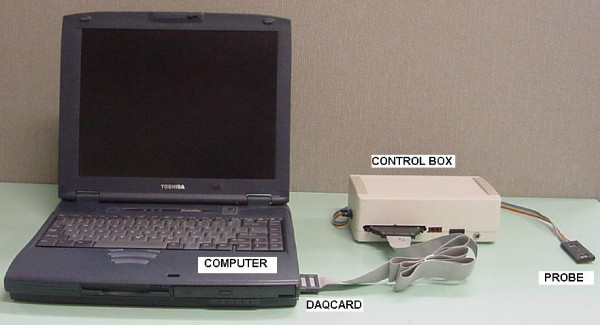
System containing computer, control box and probe

The cwNIRS system consists of three parts: probe, control circuit and processing unit (fig 2). The probe constitutes the interface between the control system and the subject. It holds the light source and detector in an appropriate geometry. Operation of the light source and detectors are manipulated by the control circuit which can be subdivided as transmitter and receiver. Transmitter and receiver are controlled by the computer software for coherent detection of two wavelengths. The computer also stores and displays received light information after applying necessary signal processing schemes.

**Figure 3:**
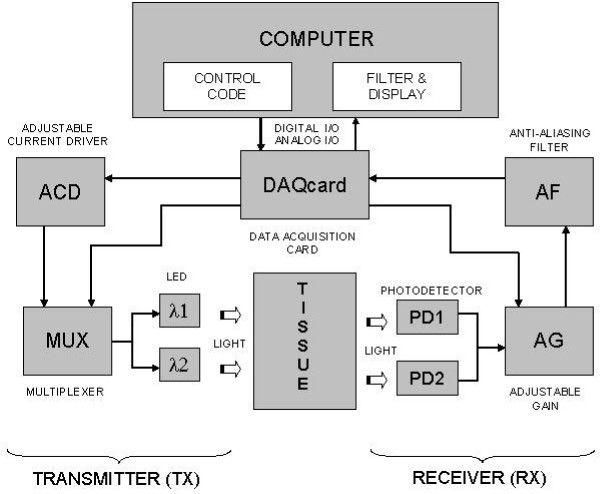
Block diagram of the cwNIRS system

The design of the system can be explained through the use of four subtitles: transmitter, probe, receiver and computer processing. The role of each part is displayed in the detailed block diagram of the system (fig 3)

##### 1. Transmitter

The transmitter part of the control circuit is composed of an adjustable LED driver and wavelength selector. The purpose of the adjustable LED driver is to regulate the light output of the LED in order to compensate for absorption differences in various tissue types. For subjects with lighter skin color, sufficient amount of output can be achieved by applying lower power light whereas larger intensity of light is necessary for darker skin color. This is because of the relative amount of melanin in skin. Since melanin concentration is constant during the measurement, it does not affect the results related to concentration change. However, darker skin color causes a decrease in signal to noise ratio (SNR) by absorbing more light. Therefore, the same level of SNR can be achieved by adjusting the intensity of NIR light.

Light intensity can be changed by varying the current passing through the light source. The user determines the amount of current and enters it into the user interface. Then, the software adjusts the current by changing the resistance of a digital potentiometer in driver circuit. The range of current that can pass through the light source is from 0 to 100 mA. Values larger than 100 mA have potential to damage the light source; therefore, the software warns the user about these values through the user-interface and does not initiate the operation. The device also has an indicator light on the electronics box which turns on to warn the user when a current larger than 100 mA passes through the light source due to an undesired short circuit.

As described in the previous section, two different wavelengths are required to resolve two types of blood chromophores. We employed these two wavelengths together by means of time multiplexing. This is implemented by a multiplexer IC which is controlled by the software. At the same time, data from corresponding detector channel were registered to satisfy coherent detection. The multiplexer was not directly connected to the light source, since current in the order of 10 to 100 mA passes through the source. Relatively higher R_ON _resistance of the multiplexer causes a large voltage drop and necessary voltage may not be supplied to light source to turn it on. Instead, we connected multiplexer to operate an analog switch with a very low resistance value to turn on/off the light source. This permits the use of conventional camcorder batteries with low voltage values to power the system.

During each cycle, after time multiplexing two wavelengths, we allow an idle period where no wavelength is turned on. The reason for this idle period is that NIR light source is a semiconductor junction and heats up during the operation. Experimentally, it has been shown that an idle time helps light source to cool down to safe ranges (see system performance part). Moreover, when NIR light is shone through the tissue, detector readings are composed of ambient light penetrating through the tissue and the offset of the electronic components in addition to optical information coming from the brain. These offset values can be determined by recording detector output when NIR wavelengths are turned off. During each cycle, detector reading for idle period is used to correct the mentioned offset values in order to increase the accuracy of the readings. Furthermore, detector output during dark period can be used to monitor the optical isolation of detectors from direct incidence of the ambient light. If offset values are larger than expected, the probe is reattached to the skin for better coupling and isolation.

##### 2. Receiver

The receiver part transfers light information obtained from detectors to the computer after amplifying and analog filtering. The aim of the amplification part is to bring the signal level close to the top of the dynamic range of the analog to digital converter. This operation minimizes the error that occurs during quantization process. The amplification process is initiated by the user. The user hits a button on the user interface after obtaining a satisfactory probe-skin coupling during calibration process. The software reads the light output coming from the brain and adjusts the value of a digital potentiometer in the gain amplifier such that the signal value is amplified to the full dynamic range. A quantization SNR of 75 dB is guaranteed as the result of closed-loop gain adjustment procedure where quantization resolution is 12 bit. The gain value is also displayed on the user-interface. Typical photon sampling frequency is 60 kHz and wavelength multiplexing frequency is 10 Hz.

Before digital conversion, the signal is low-pass filtered with cutoff frequency of 1.5 kHz to avoid aliasing during the sampling process.

##### 3. Probe

The probe is the most critical part of the system design since it establishes the interface between electronics and the subject. It holds the light source and two detectors in an appropriate geometry.

**Figure 4:**
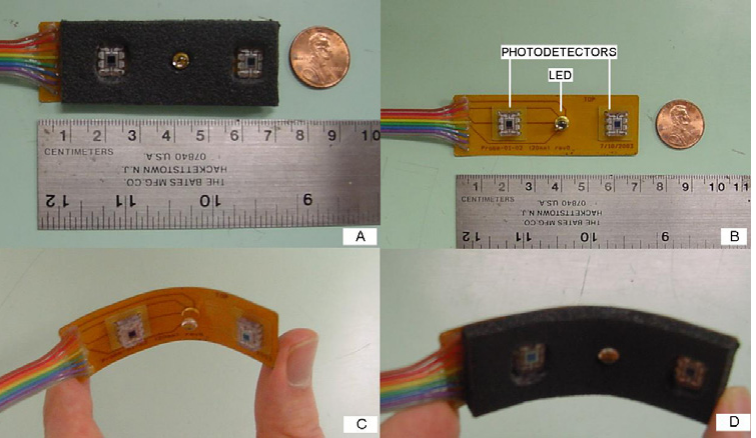
The first prototype of the "flexible" probe holding low power LED and detectors

Detectors are positioned on opposite sides of the source with a source-detector distance of 2 cm as seen in fig 4. This geometry allows monitoring of two different locations of the brain, simultaneously. Approximate penetration depth is 1 cm for this configuration [2]. Alternatively, detectors can be located on the same side with different source-detector distances for multi-depth measurements. By monitoring different depths, absolute saturation of the brain tissue can be detected.

In our research, we prefer light emitting diode (LED) as the source of the NIR light instead of using laser. Higher light intensity levels can be utilized with LED, since it is a non-coherent and non-collimated light source. Power consumption of LED is minimal with respect to the laser source, hence the system can be battery operated. Laser light source also suffers from extreme heating of the semiconductor junction. Optical fibers are required to carry laser light to tissue.

Therefore, more instrument space is needed to provide cooling. Furthermore, laser source needs additional precautions due to its injury potential on the eye. It may not be appropriate for use in neonatal intensive care units for vulnerable population of newborns. As a result, practical and portable systems are quite difficult and inappropriate to build with laser source.

Single package LEDs which contain 2–3 wavelengths together are available in the market (e.g. Epitex, Inc). We selected a source where 730 and 850 nm are enclosed in a TO18 package. Crosstalk between these two wavelengths is less significant with respect to other wavelength pairs[21]. This combination of wavelengths is appropriate for our purpose of resolving two chromophore concentrations. LED can emit output power between 3 and 17 mW where the typical power used is 9 mW. The spectrum purity of this LED in the NIR region is around 30 nm. Although it is wider with respect to diode lasers (5–6 nm), 30 nm gives enough sharpness to resolve two wavelengths since absorption spectra is relatively flat around source wavelengths.

Monolithic photodiode/preamplifier integrated circuits housed in a clear 8-pin DIP package (OPT101, Burr-Brown^®^, Co.) were used to detect the light coming from the brain. Active sensing area of this detector is 2.29 × 2.29 mm^2^. Feedback resistance of 1 Mohm provides a transimpedance (V/A) gain of 106 and bandwidth more than 100 Hz.

The most challenging aspect of the design has been the coupling of optical elements (optodes) to the scalp of the subject. We have used flexible circuit technology to fit the optodes to the curved surface of the subject's head. The source and detectors attached to the flexible circuit board are embedded into medically graded foam (fig 4). This prevents relatively sharp corners of optodes to come in contact with the scalp of the subject. Moreover, it blocks the ambient light which tends to saturate the photodiodes.

**Figure 5:**
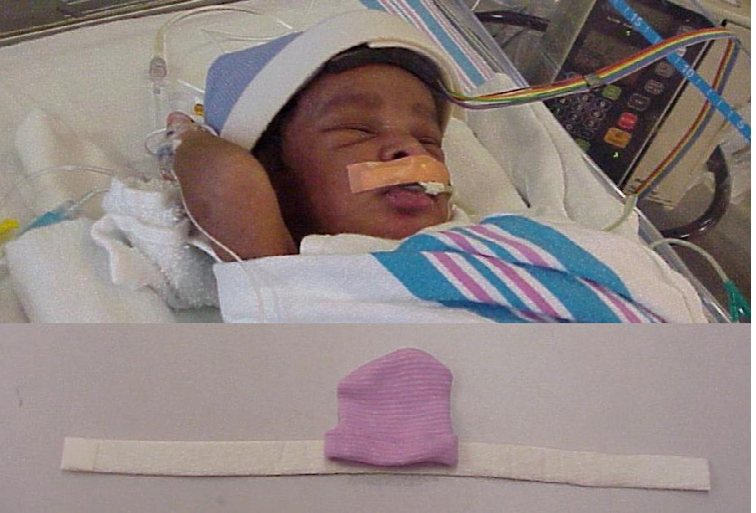
Baby hat method used for optode-scalp coupling (see "Acknowledgment")

We developed various methods to affix the flexible probe to the surface of the head. The first method is to use double sided medically graded sticky tape with one side attached to the foam and other side attached to the scalp (Adchem^®^). This provides a satisfactory and stable optode-scalp coupling. Another method uses a medically graded self-sticky silicon material (Implantech, Inc) instead of sticky tape and the foam; optodes can easily be embedded in this silicon material. In yet another method, we used medically graded foam again. In this case, stable optode-scalp coupling is provided by inserting the foam under a baby hat as in fig 5. This baby hat is generally utilized in NICU to hold incubator tubes running to newborn's nose and mouth. The performances of these methods were similar in terms of ease-of-use and coupling efficiency.

In order to avoid the effect of ambient light further and to increase the SNR, we attached an NIR filter (Edmund Optics^®^) with cut-off wavelength of 700 nm to the surface of the photodiode. This introduced an insulation layer between photodiodes and scalp, in addition to rejecting non-NIR wavelengths.

An 1850 mAh Li-Ion camcorder battery with 7.2 V voltage supply was used to power the transmitter, receiver and the probe. A charging system was also added to the box to charge the battery without taking it out of the box.

##### 4. Processing Unit

A data acquisition card with 10 V dynamic range and 12-bit resolution (DAQcard 1200, National Instruments™) has been used to convert analog information to digital where sampling rates up to 100 kHz can be achieved. 5 digital output and 2 analog input channels of the data acquisition card have been sufficient for the entire operation of the system.

The system hardware is software (visual C++^®^) operated: the custom software manages time multiplexing operation between source and detector pairs, the coherent detection from multiple sensors and incorporation of real time digital signal processing algorithms. The user interface software displays changes in blood volume, oxygenation and, concentrations of Hb and HbO_2 _by implementing Beer-Lambert Law in real time. In order to monitor optode-scalp coupling during the operation, the user interface also displays raw voltage data coming from control box. Especially, the offset value read during the idle period of the light source is an important parameter demonstrating the ambient light leakage. Data is recorded in a file for further off-line processing.

**Figure 6:**
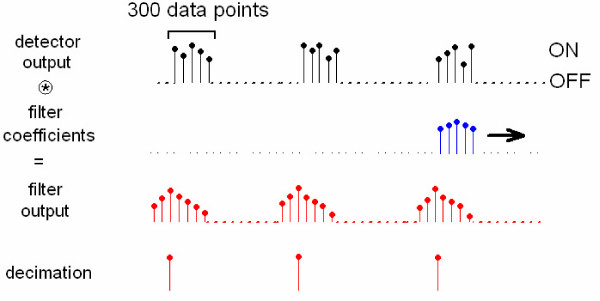
Summary of the filtering operation

Typical time multiplexed voltage output of the detectors can be represented as seen in fig 6. Each square pulse corresponds to detected photons when a particular wavelength is turned on. This signal can be interpreted as a low-pass signal multiplied by a train of square pulses, which corresponds to convolution with sinc functions in frequency domain. Thus, processing of time multiplexed signal is not straightforward. However, we can get rid of time multiplexing scheme by applying finite impulse response (FIR) filtering followed by decimation. As we convolve FIR filter with detector output, we only record the result of the convolution when all FIR filter coefficients coincides with the samples of the square pulse as in fig 6. To have a sharper cut-off, filter order is selected to be equal to the number of samples that each square pulse contains. We designed an FIR filter with 10 Hz cut-off frequency by using Dolph-Chebyshev window function with a ripple factor of 40. The operation of FIR filtering and decimation are applied real time by the control software.

After decimation process, an additional FIR low-pass filter is applied to the signal to clean various artifacts. The NIRS signal that corresponds to hemodynamic activity of the brain has a very slow time course[15]. The expected frequency range of the hemodynamic activity is 0.06–0.16 Hz interval. On the other hand, hemodynamic activity signal is contaminated by other factors such as respiration, arterial pulsations and motion artifacts. Fortunately, all these artifacts are in higher frequency ranges with respect to hemodynamic activity. An online FIR filter is used to clean the frequencies above 0.16 Hz which is out of region of interest. Unfiltered data is recorded in parallel to filtered data for further offline-processing.

## III. Results

### A. System Performance

The performance of the device was evaluated by means of initial laboratory tests on phantom. First, shot limited signal to noise ratio (SNR) was measured. Shot noise is due to the random arrival of photons in the photodiode. Since the cause of shot noise is the quantum nature of light, it is impossible to remove it. To assess the shot noise limited SNR, we used an optical phantom (*μ*a = 0.01 and *μ*s' = 1.00 mm^-1^), which is free of motion artifact and physiological noise.

We measured SNR for various values of time multiplexing parameters: duty ratio and pulse repetition rates. SNR depends on these factors in two ways. First, duty cycle and pulse repetition rate determine the number of collected data points. More number of data points for each pulse is supposed to provide an increased SNR since the performance of the FIR filter is improved. On the other hand, duty cycle and pulse repetition rate affect the heating of the semiconductor junction of the LED. Longer duty cycle and pulse repetition rate cause an elevation in the temperature of the semiconductor junction. Temperature increase adds an extra noise to the generated light output, thereby, significantly limiting the SNR. Furthermore, this is an undesired condition since elevated temperature is a risk for the safety of the device on subject population. Therefore, we face a trade-off between filter performance and temperature rise of the semiconductor junction for duty ratio and pulse repetition rate to determine SNR.

**Figure 7:**
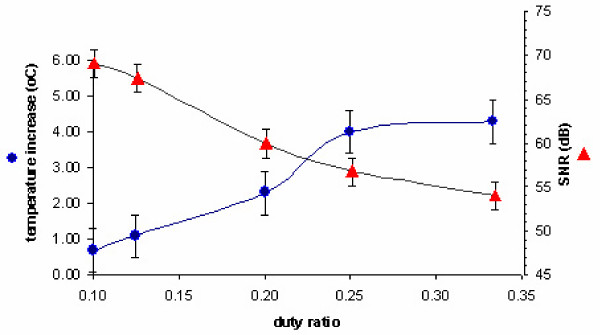
Temperature increase & SNR vs. pulse repetition rate (duty ratio = 12.5%)

**Figure 8:**
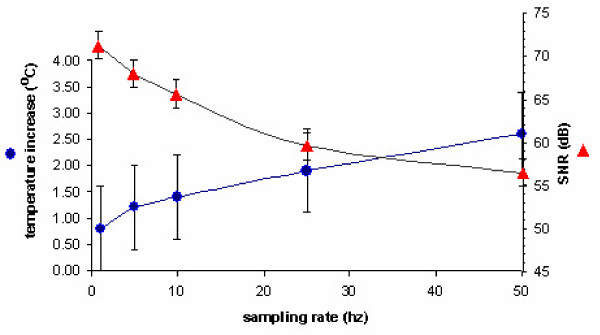
Temperature increase & SNR vs. duty ratio (pulse repetition rate = 10 Hz)

The experimental results of temperature increase and SNR values for different duty ratios and pulse repetition rates are presented in fig 7 and 8. 60 kHz of sampling rate has been used during all measurements and temperature value at the 30th minute of the operation has been taken when temperature asymptotically converges to a stable value. Increases in both pulse repetition rate and duty cycle caused a temperature increase in the semiconductor junction. The expected rise in SNR due to increased number of samples is suppressed by extra noise in the light output of the LED as result of temperature increase. So, overall SNR of the device decreased.

As a result of this analysis, operation duty cycle and pulse repetition rate were selected to be 12.5% and 10 Hz, respectively. This results in a relatively small amount of temperature increase whereas a SNR of 67 dB was obtained. Such high values of SNR could be achieved as a result of powering the entire system including the control box and processing unit by battery. An off-the-shelf 1850 mAh Lithium-Ion battery operates the system for more than 30 hours.

Temperature increase is an important factor to evaluate the safety of the system. NIR light is a non-ionizing type of light and its only known potential hazard is thermal injury of the skin. There are two factors that contribute to the temperature increase of the skin: radiated heat due to NIR light absorption by the skin and conducted heat due to heating of semiconductor junctions inside the LED, since LED is attached to the skin. Our previous study[22] demonstrates that temperature increase due to radiated heat is around 0.5–1°C, whereas, temperature increase due to conducted heat can be as high as 10°C. On the other hand, the heating of the semiconductor junction can be controlled by adjusting the pulse repetition rate and duty cycle, therefore, the effect of conductive heat can be minimized. When above proposed pulse repetition rate and duty cycle are used, the combined temperature increase of the skin is around 1.5°C and has no harmful effect to the subject under normal conditions. Therefore, the risk of the system for any potential burn injury is minimal.

Stray light rejection has found to be larger than 99% under normal illumination levels. This has been increased even more with the use of NIR filter. Inter-channel crosstalk between detectors was measured to be less than 0.1%.

### B. Preliminary Evaluation

#### a. Liquid phantom experiment

A dynamic liquid phantom simulating the optical properties of the tissue was used in order to test the efficacy of the system. The basis of the phantom was formed by a scattering solution of Intralipid (Liposyn III) and phosphate buffered saline with pH = 7.4. 27.2 ml of intralipid was added to 1000 ml of water in order to obtain an overall reduced scattering coefficient of 0.8 mm^-1 ^(730 nm). The solution was placed in a cylindrical beaker. A magnetic stirring rod was also placed in the beaker and the phantom was stirred during the course of the experiment. After the basis was formed, red blood cells obtained from healthy human blood were added to scattering solution to achieve a volume fraction of 1.5% and total hemoglobin concentration of 26 *μ*M. This is a typical value for normal physiological conditions with an assumption of 4.0% blood volume and 40% hematocrit. The hemoglobin saturation was measured to be 85%.

In order to induce deoxygenation on the liquid phantom, 4 g of dry Bakers yeast was added to the solution. The temperature of the phantom was maintained at 37°C to keep the yeast active. This condition was maintained over a course of about 20–30 min. Deoxygenation was observed until hemoglobin saturation reached a steady state at 25%. After deoxygenation of yeast-intralipid solution reached steady state, oxygenation was simulated again by delivering extra oxygen to the phantom from an oxygen tank. Oxygen supply was maintained until a steady state level of oxygenation was obtained. Steady state hemoglobin saturation was 89%.

**Figure 9:**
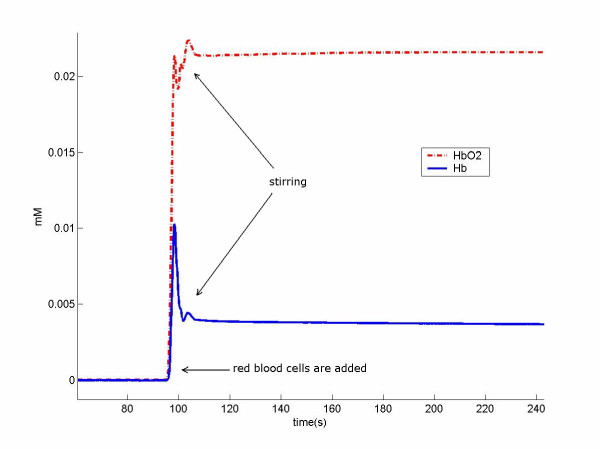
Changes in hemoglobin concentration during red blood cell addition

During this procedure, two sets of data were collected by attaching the cwNIRS probe to the side of the beaker. The first measurement was performed to observe the effect of HbO_2 _and Hb addition to the intralipid solution. Since there were no hemoglobin molecules in the intralipid solution, this measurement provided the absolute concentrations of added HbO_2 _and Hb as seen in figure 9. In this figure, the ratio of HbO_2 _and Hb is consistent with the measured saturation value of 85%.

**Figure 10:**
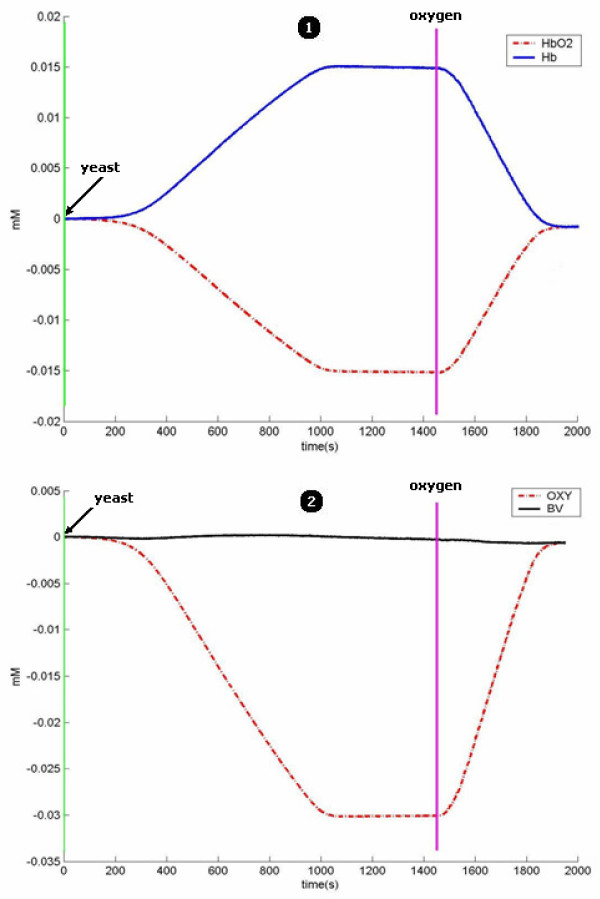
Changes in hemoglobin concentration (1), OXY and BV values (2) during the yeast test

In the second measurement, our aim was to observe deoxygenation as a result of yeast addition. Here, the baseline was taken as the steady state intralipid-hemoglobin solution without yeast. Yeast activation related changes in HbO_2 _and Hb concentrations can be seen in figure 10 in addition to BV and OXY curves. When yeast was added to the solution, deoxygenation was due to the consumption of oxygen by yeast which reached steady state within 15 minutes. Steady state values are consistent with the measured saturation of 25%. Re-oxygenation, when extra oxygen was supplied, is displayed in the same figure. During the procedure of deoxygenation and oxygenation, as expected, blood volume did not change significantly.

#### b. Monitoring of newborn subjects

The validity of the system was tested on a newborn in NICU of St. Peter's University Hospital (NJ) by monitoring oxygenated and deoxygenated hemoglobin concentrations on the temporal region of the brain as a response to a standard auditory brainstem response (ABR) based hearing screening test. A hearing screening test, which is mandatory by law in many states, is applied to newborns to screen for hearing problems. In our experimental protocol, we used hearing screening test to create a controlled auditory stimulation in the temporal region of the infant brain. Local temporal activation has been observed as a response to auditory stimuli in fMRI studies in the literature[23].

All studies were carried out under an IRB approved protocol and informed consent was obtained prior to studies. In this study, a five day old 37 week estimated gestational age male infant underwent an ABR based hearing screening test while he was being monitored by the cwNIRS system. In an ABR based hearing screening test, EEG electrodes are attached with adhesive to the baby's scalp. While the baby sleeps, clicking sounds are applied through tiny earphones in the baby's ears. The test measures the brain's electrical activity in response to the sounds and displays the result as "passed" or "failed". The noninvasive procedure takes only a few minutes.

In our study, the hearing screening test was repeated twice. During the first test, the probe was placed on the temporal region of the brain which corresponds to the auditory cortex. In the second test, the probe was located on the forehead of the newborn for the control measurement (fig. 5). Baseline data were collected for 20 seconds before each test. After baseline, a hearing screening device from Natus^®^, Inc. (ALGO 3^®^) was used for auditory stimulation by applying 34 clicks per second until the infant passed the test. Both tests lasted approximately 2 minutes. The ALGO 3^® ^hearing screening system displayed the results as "passed" for both tests which confirmed that we created auditory stimuli successfully. The entire procedure was repeated twice to validate the self-consistency of the data.

**Figure 11:**
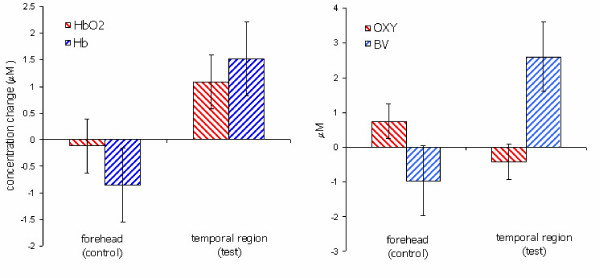
Average changes in hemodynamic parameters in response to hearing screening test

Average changes in the hemoglobin concentrations in addition to OXY and BV signals during stimulation with respect to baseline period are presented in figure 11. Error bars indicate the results of two successive experiments. An increase in both oxygenated and deoxygenated hemoglobin concentrations was observed in the temporal (test) region during auditory stimulation. Observed increase in blood volume was a result of blood rush to the local tissue. On the other hand, average deoxyhemoglobin concentration change was bigger than oxyhemoglobin concentration which was an indication of oxygen consumption as a result of the local activity. In the forehead (control) probe, only small fluctuations around the baseline were recorded which were uncorrelated with the stimulation. Observed changes are consistent with other NIRS based neonatal measurements in the literature [10,11].

## IV. Conclusion

We present the design of a low-cost, battery operated continuous wave NIRS system that is intended for continuous bedside brain hemodynamic monitoring of newborns in neonatal intensive care units. Such a system can potentially assist clinicians in assessing functional changes in cerebral oxygenation and blood volume without removing the baby from the NICU.

Design parameters were defined and optimized for a safe and effective system performance. The most critical design issue was the trade-off between the temperature of the LED and the performance of the digital filter. SNR of 67 dB was obtained for a temporal resolution of 10 Hz. The system can be operated for 30 hours with an off-the-shelf 1850 mAh Li-Ion battery. Stray light rejection was satisfactory and inter-channel crosstalk of the channels was less than 0.1%.

Preliminary experiments performed both in the laboratory and in a clinical setting suggest that the system can be used to track functional changes of blood volume and oxygenation. This system can be used for bedside monitoring of neonates undergoing various clinical studies such as apnea, asphyxia, hypoglycemia, endotracheal suctioning, surfactant, aminophylline and indomethacin administrations.

## Authors' contributions

AB and HR carried out the in-vivo and in-vitro experiments and AB drafted the manuscript. BO and AR conceived of the study, and participated in its design and coordination. All authors read and approved the final manuscript
